# Phosphorylation in the Ser/Arg-rich region of the nucleocapsid of SARS-CoV-2 regulates phase separation by inhibiting self-association of a distant helix

**DOI:** 10.1016/j.jbc.2024.107354

**Published:** 2024-05-07

**Authors:** Hannah Stuwe, Patrick N. Reardon, Zhen Yu, Sahana Shah, Kaitlyn Hughes, Elisar J. Barbar

**Affiliations:** 1 Department of Biochemistry and Biophysics, Oregon State University, Corvallis, Oregon, USA; 2 OSU NMR Facility, Oregon State University, Corvallis, Oregon, USA

**Keywords:** SARS-CoV-2, phosphorylation, AUC, NMR, LLPS, protein RNA interactions

## Abstract

The nucleocapsid protein (N) of SARS-CoV-2 is essential for virus replication, genome packaging, evading host immunity, and virus maturation. N is a multidomain protein composed of an independently folded monomeric N-terminal domain that is the primary site for RNA binding and a dimeric C-terminal domain that is essential for efficient phase separation and condensate formation with RNA. The domains are separated by a disordered Ser/Arg-rich region preceding a self-associating Leu-rich helix. Phosphorylation in the Ser/Arg region in infected cells decreases the viscosity of N:RNA condensates promoting viral replication and host immune evasion. The molecular level effect of phosphorylation, however, is missing from our current understanding. Using NMR spectroscopy and analytical ultracentrifugation, we show that phosphorylation destabilizes the self-associating Leu-rich helix 30 amino-acids distant from the phosphorylation site. NMR and gel shift assays demonstrate that RNA binding by the linker is dampened by phosphorylation, whereas RNA binding to the full-length protein is not significantly affected presumably due to retained strong interactions with the primary RNA-binding domain. Introducing a switchable self-associating domain to replace the Leu-rich helix confirms the importance of linker self-association to droplet formation and suggests that phosphorylation not only increases solubility of the positively charged elongated Ser/Arg region as observed in other RNA-binding proteins but can also inhibit self-association of the Leu-rich helix. These data highlight the effect of phosphorylation both at local sites and at a distant self-associating hydrophobic helix in regulating liquid–liquid phase separation of the entire protein.

SARS-CoV-2 is an enveloped single-stranded, positive sense RNA virus with a large 30kb genome (1). The SARS-CoV-2 virion is composed of four structural proteins: spike (S), membrane (M), envelope (E), and nucleocapsid (N). The viral membrane, with S, M, and E, surround the helical nucleocapsid containing the viral RNA genome encapsulated by N (1, 2, 3). N has several functions within the viral life cycle but is primarily involved in protecting the viral RNA genome by binding, condensing, and packaging it within the virion (2, 4). N also functions in the replicase transcriptase complexes where it mediates the synthesis of genomic RNA (gRNA) within the host cell (5, 6, 7). Additionally, N contributes to innate immune evasion *via* sequestering stress granule protein G3BP1 (8, 9). N’s ability to phase separate, particularly with gRNA, has been extensively investigated (3, 4, 10, 11, 12, 13, 14, 15, 16, 17, 18) and undergoing this process is what allows N to participate in a vast and varied functions (12, 19). These N-containing condensates are also a promising target for drug developments (20).

N is a 419 amino-acid long protein with two independently folded domains, the N-terminal domain (NTD) and the C-terminal domain (CTD) (Fig. 1) (21). These domains are flanked by two disordered tails and separated by a central disordered linker that contains a serine/arginine rich (SR-rich) region, followed by a short Leu-rich helix (LRH) (Fig. 1). The CTD forms a domain swapped dimer, facilitating the dimerization of N (21, 22, 23) and is essential for phase separation with RNA (24) while the NTD is the primary RNA-binding domain (25, 26, 27, 28) and its binding to RNA is strengthened in constructs that contain the intrinsically disordered N-terminal and central regions (29, 30). Upon binding to RNA, the N protein packs the NTD and the CTD closer together (31). Multiple studies using hydrogen-deuterium exchange mass spectrometry and analytical ultracentrifugation have shown that the central linker self-associates (32, 33) and using molecular dynamics modeling, have predicted the helical LRH region as the most likely site of self-association (33, 34, 35).

**Figure 1:**
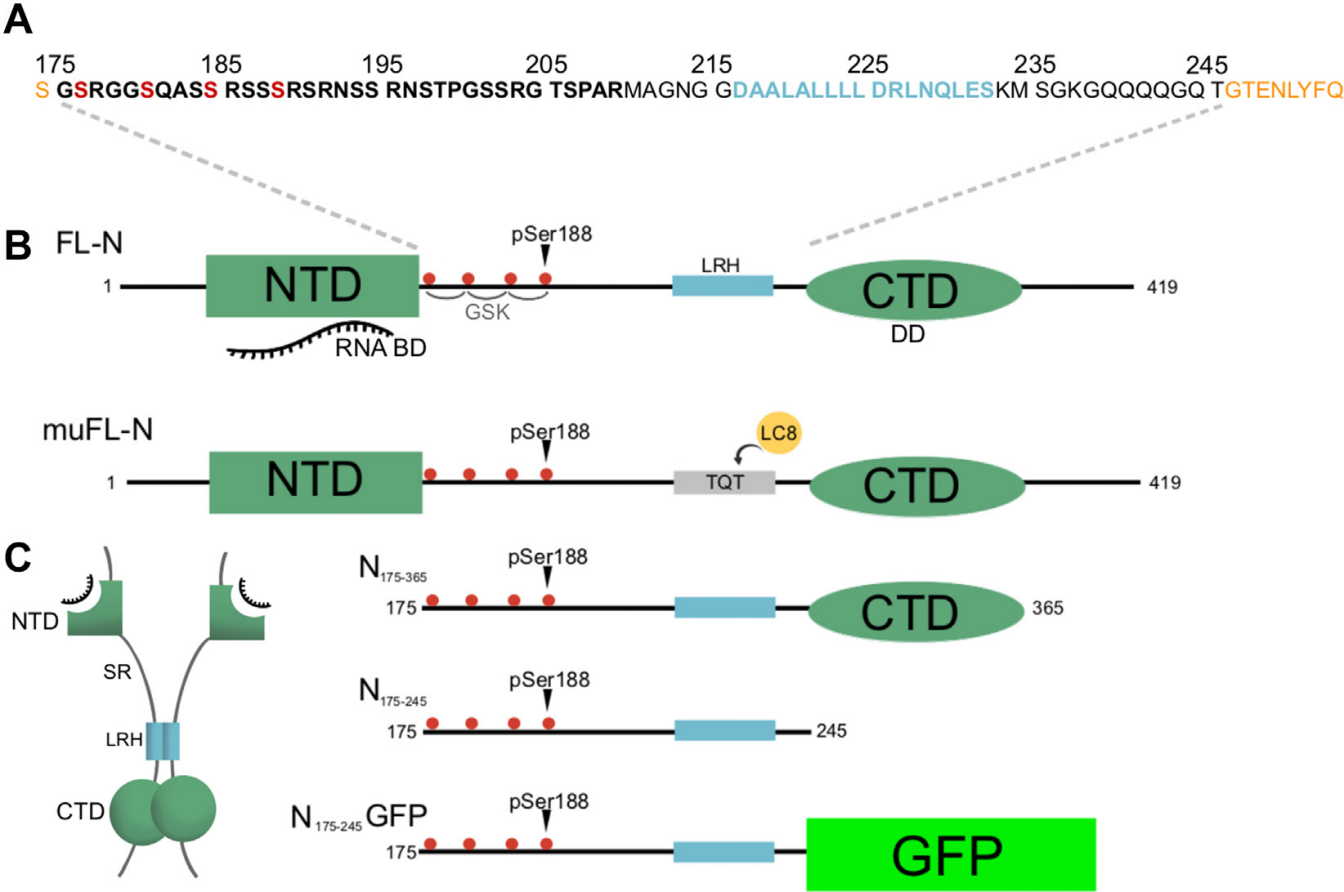
**Domain maps of SARS-CoV-2N and constructs used in this work.***A*, amino acid sequence of SARS-CoV-2N construct spanning the linker region from residues 175 to 245. Residual TEV protease cleavage sites are in *orange*. The SR-rich region is in *bold black*, with phosphorylation sites investigated here in *bold red*. The Leu-rich helix (LRH) spanning residues 216 to 232 is in *bold blue*. *B*, domain maps of full-length (FL-N), mutant full-length (muFL-N), shorter constructs N_175–365_, N_175–245_, and GFP-tagged N_175–245_ from *top* to *bottom*, respectively. Intrinsically disordered regions are represented by *black lines*, the structured RNA-binding domain NTD is represented by a *dark green rectangle*, and the dimerization domain CTD by a *dark green oval*. The LRH is represented by a *small blue rectangle*. GSK priming site pSer188 is pointed to by an *arrow*, and the subsequent phosphorylation sites are indicated by *red circles*. The muFL-N has a TQT recognition motif for LC8-binding site incorporated to replace the LRH shown as *gray rectangle*. *C*, a cartoon depiction of dimeric FL-N showing self-association of LRH- and RNA-binding site of the NTD. CTD, C-terminal domain; GSK, glycogen synthase kinase; LC8, dynein light chain 8; LRH, Leu-rich helix; NTD, N-terminal domain; SR-rich, serine/arginine-rich.

The dual roles of N in the assembly with gRNA into viral RNA-protein complexes and in localization to the replicase transcriptase complexes to enhance viral replication and transcription are regulated by phosphorylation of the SR-rich region of N (36, 37, 38, 39). Phosphorylation of the homolog SARS-CoV-1 SR-rich region did not significantly affect RNA binding of the nucleocapsid protein but impaired its ability to form oligomers (40). In SARS-CoV-2, phosphorylation of N altered liquid-liquid phase separation (LLPS) formation, with unmodified protein forming gel-like condensates, while phosphorylated protein forming more dynamic liquid-like droplets (19). In a follow up study, the Morgan group showed that the assembly into viral particles requires the LRH and using phosphomimetic SR demonstrated that phosphorylation inhibits vRNP assembly (41). The phosphorylation-mediated differences in LLPS characteristics are hypothesized to contribute to N switching between functions, with liquid-like phosphorylated N droplets potentially promoting viral replication and host immune evasion and gel-like unphosphorylated droplets promoting viral RNA packaging (10, 11). Overall, these observations demonstrate the critical role of phosphorylation in regulating the function of N during the virus life cycle. It is notable that the SR-rich region in the linker can have up to 15 phosphorylation sites depending on cell type (42) which will presumably alter the structure and interactions of the highly positive charge pattern of the unphosphorylated protein.

Here, we systematically probe the effect of a single phosphorylation event, at serine 188, a known priming site of phosphorylation (42) and of hyperphosphorylation, *via* glycogen synthase kinase 3β (GSK-3), on the structure of the linker domain, the self-association of the LRH, and the interactions of the full-length protein with gRNA, including its ability to phase separate. Our structural and functional characterization demonstrate the importance of phosphorylation in affecting not only the local environment but also in inhibiting the self-association of LRH distant from the site of phosphorylation. We expect that phosphorylation at all potential sites will cause an even more profound change. We present a model that explains how phosphorylation could alter the structure of N in condensates and force unpackaging of viral RNA.

## Results

### Association of the central linker is localized to helical residues 216 to 232

**Figure 2:**
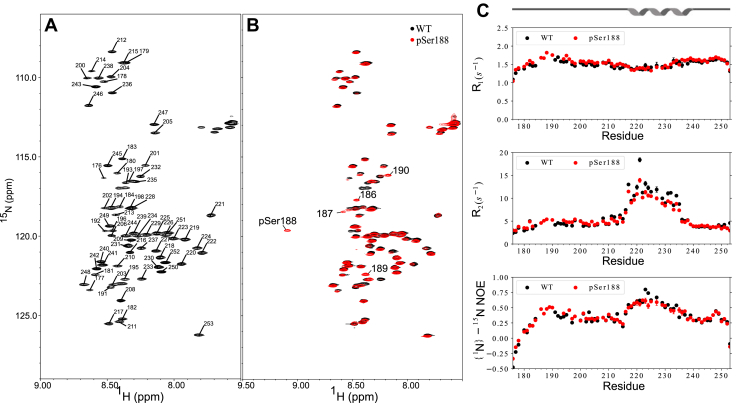
**NMR structural characterization of WT N175 to 245 and pSer188 N175 to 245.***A*, ^15^N-HSQC spectrum of 150 μM WT N_175–245_ at 10 °C with backbone resonance assignments. *B*, overlay of ^15^N-HSQC spectrum of WT N_175–245_ (*black*) and pSer188 N_175–245_ (*red*), with shifted peaks labeled. *C*, ^15^N nuclear spin relaxation of WT N_175–245_ (*black*) compared to pSer188 N_175–245_ (*red*). The Leu-rich helix is indicated.

A suite of BEST triple resonance NMR experiments helped assign the backbone resonances of N_175–245_ at 10 °C (Fig. 2), including the 225 to 230 region missing in the assignments deposited in the BMRB (43). The ^15^N HSQC spectrum is consistent with a primarily disordered polypeptide, with generally narrow chemical shift dispersion. Fast time scale dynamics determined by measuring nuclear spin relaxation ^15^N R_1_, ^15^N R_2_, and {^1^H}-^15^N Nuclear Overhauser Effect (NOE) identified residues 178 to 215 and 236 to 248 to be fully disordered with NOE’s below 0.5 and negative NOE’s at the termini (Fig. 2). ^15^N R_2_ and R_1_ relaxation rates in these regions were relatively uniform, while ^15^N R_2_ and {^1^H}-^15^N NOE were elevated for residues 216 to 232, which together with the carbon chemical shifts (Fig. S1) confirm that residues 216 to 232 adopt a helical structure and that except for this short helix, N_175–245_ does not adopt any regular secondary structure under our experimental conditions.

**Figure 3:**
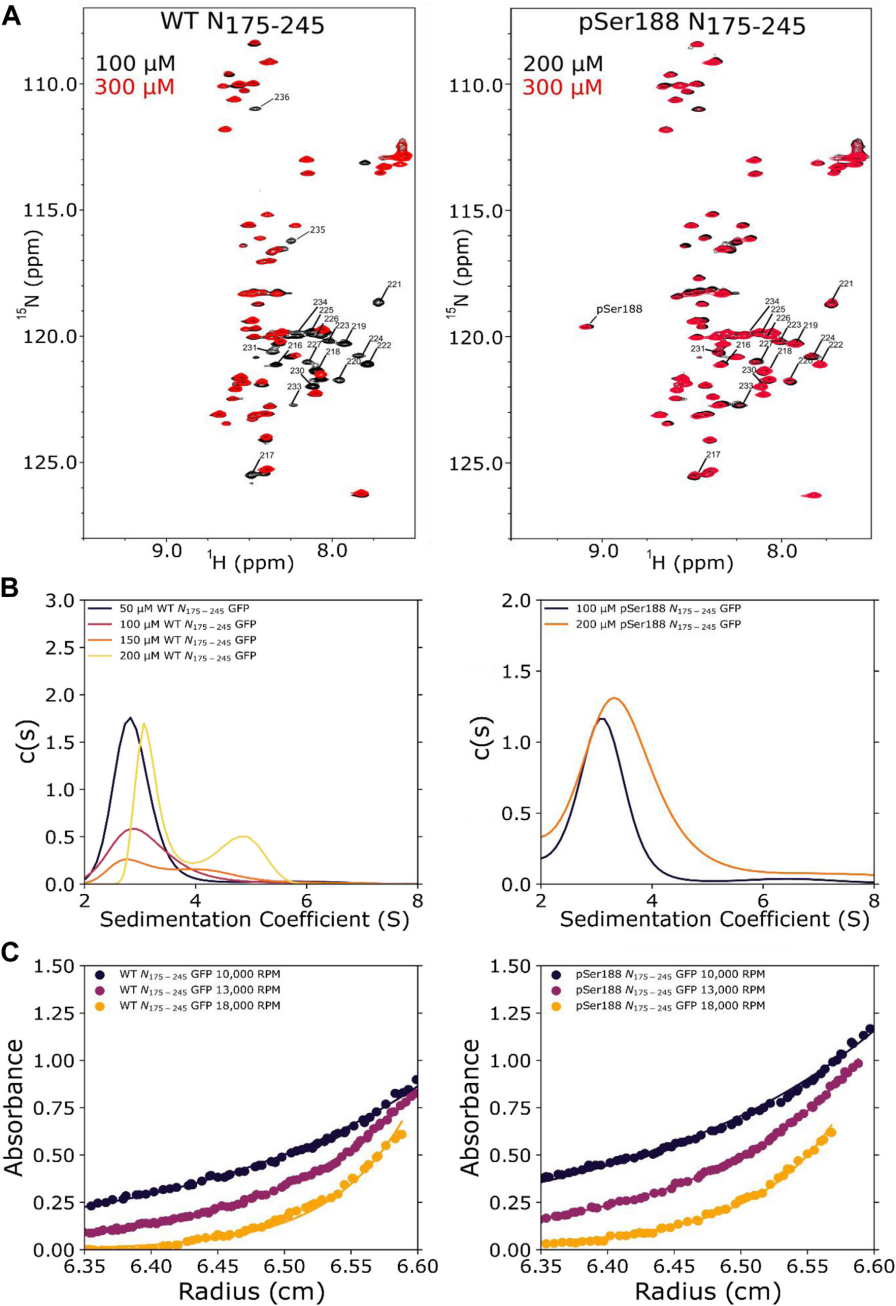
**Concentration dependence of self-association of WT and pSer188 N175 to 245.***A*, ^15^N-HSQC spectra of WT N_175–245_ (*left*) at 300 μM (*red*) and 100 μM (*black*) concentration. Resonances that disappear at higher concentration are labeled. ^15^N-HSQC spectra of pSer188 N_175–245_ (*right*) at 300 μM (*red*) and 200 μM (*black*) concentration. The same resonances are labeled. Missing peaks for residues 233 to 235 is due to ordered structure in proximity of the self-associating helix. *B*, sedimentation velocity analytical ultracentrifugation (SV-AUC) of WT-N_175–245_ GFP (*left*) at 50 to 200 μM concentration and SV-AUC of pSer188 N_175–245_ GFP (*right*) at 100 and 200 μM concentration. *C*, representative sedimentation equilibrium data at three rotor speeds for WT-N_175–245_ GFP (*left*) and for pSer 188 N_175–245_ GFP (*right*). Monomer-dimer equilibrium fits are shown as *solid* lines. All AUC experiments with N_175–245_ were performed with GFP fusion.

As stated above, missing peaks for the deposited assignments were attributed to self-association of the central linker (43). Consistent with this interpretation, the peaks assigned at lower concentrations of ^15^N-labeled N_175–245_ (Fig. 3) correspond to the helical region and thus identify self-association to be localized to the helical residues. At 300 μM, these peaks disappeared from the spectrum (Fig. 3).

To confirm that peak attenuation is in fact due to self-association and to evaluate the strength of self-association, we performed sedimentation velocity analytical ultracentrifugation (SV-AUC) experiments on GFP-tagged N_175–245_ (WT-N_175–245_ GFP). The GFP tag was used to provide a stronger UV absorbance. The SV-AUC profiles clearly show two peaks in the WT- N_175–245_ GFP at 200 μM, corresponding to ∼3.1S and 4.8S, suggesting the presence of monomeric and dimeric species (Fig. 3). The GFP control at the same concentration only gave rise to a single peak, indicating no significant dimerization under these conditions (Fig. S2). Subsequent sedimentation equilibrium analysis (SE-AUC) gave a mass average molecular weight for N_175–245_ GFP of ∼66 kDa near the expected molecular weight of 72 kDa for a dimer (Fig. 3) which upon analysis to a monomer-dimer equilibrium model yielded a dissociation constant of 77 μM. Together, these data indicate that the LRH can self-associate but only weakly in the linker construct.

### Self-association of LRH is strengthened in context of dimeric CTD

**Figure 4:**
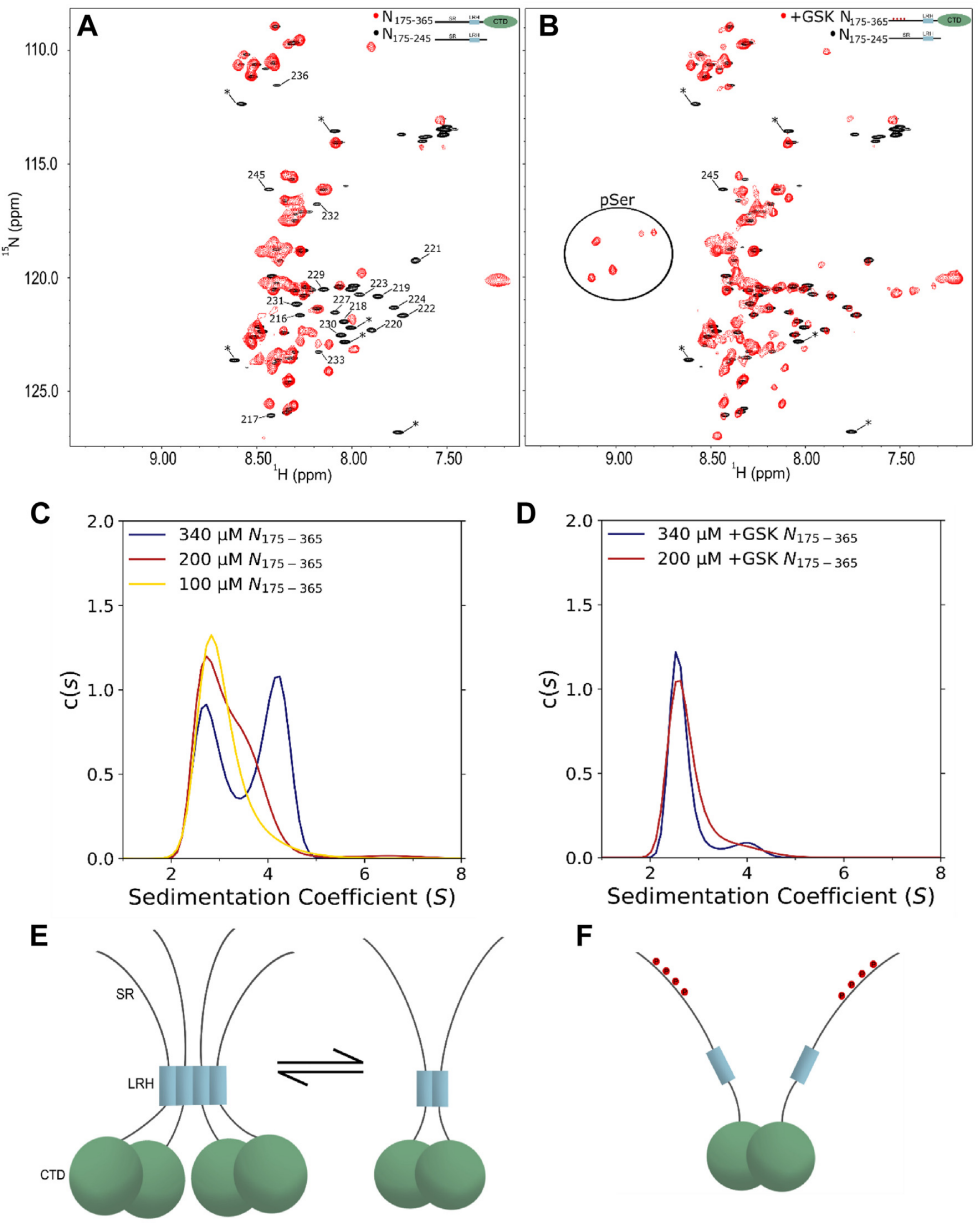
**Concentration dependence self-association of WT and phosphorylated N175 to 365.***A*, ^15^N-TROSY-HSQC spectra of 50 μM WT N_175–365_ (*red*) compared to WT N_175–245_ (*black*). Resonances of N_175–245_ that are not present in N_175–365_ are labeled. Resonances that correspond to residual TEV cleavage sites in N_175–245_ are indicated by an *asterisk*. *B*, ^15^N-TROSY-HSQC spectra of 50 μM GSK-hyperphosphorylated N_175–365_ (+GSK N_175–365_) (*red*) compared to WT N_175–245_ (*black*). Down-field resonances corresponding to phosphorylated serine are circled. Resonance labeling scheme is the same as in (*A*). *C*, SV-AUC of WT-N_175–365_ at 100 to 340 μM concentration. *D*, SV-AUC of +GSK N_175–365_ at 200 and 340 μM concentration. *E*, representative model of proposed tetramerization of N_175–365_ due to LRH self-association (*dark blue*). Tetrameric N_175–365_ is in exchange with dimeric N_175–365_ as indicated on the *right*. *F*, representative model of the effect of hyperphosphorylation of N_175–365_ showing the CTD as an intact dimer (*green spheres*) but with dissociation of the LRH from tetramers and dimers to monomers. CTD, C-terminal domain; GSK, glycogen synthase kinase 3β; LRH, Leu-rich helix.

To probe the behavior of the LRH in the context of the full-length protein (FL-N), we collected a series of ^15^N HSQC experiments at decreasing concentrations of a ^15^N-labeled construct of N including the central linker and the CTD, spanning residues 175 to 365 (N_175–365_) as a more tractable model of dimeric FL-N. For concentrations of N_175–365_ as low as 10 μM, which is still considerably above the 1 μM dimerization constant of the CTD without the LRH (22), but significantly lower than the 77 μM dimerization constant of the LRH alone, resonances corresponding to residues 216 to 232 were still not detectable, indicating they are involved in self-association at those concentrations (Fig. 4). For comparison, these residues were observed at 200 μM in the linker construct. Additionally, SV-AUC experiments at 350 μM, the highest concentration assessed, showed two distinct peaks corresponding to ∼2.7S and 4.2S, suggesting the presence of dimeric and tetrameric species (Fig. 4). At 200 μM, only a primary peak at ∼2.7S and a shoulder in the ∼3.3 to 4.5S range were observed. This broad shoulder suggests exchange between dimeric and tetrameric states. At 100 μM, a single broad peak centering at ∼2.7S was observed, indicative of a dimeric N_175–365_ as the primary species in solution, but still undergoing exchange with higher order species. SE-AUC data collected to confirm that the higher order species is indeed a tetramer were fit to a dimer-tetramer model yielding an equilibrium dissociation constant of about 300 μM. Together, these data demonstrate that the self-association of the LRH is much stronger in context of the dimeric CTD and could form tetramers bridging two dimeric N molecules.

### Phosphorylation in the SR-rich region inhibits self-association of the LRH

N protein phosphorylation is a complex process involving several host kinases acting sequentially to phosphorylate the SR-rich region (36, 42, 44). We simplify this process by focusing only on Ser188 as an activating primer for GSK-3β, which phosphorylates the SR-rich region in an n-4 pattern. Based on the expected pattern of GSK-3 phosphorylation, with only Ser188 primed, we can expect a total of four phosphates incorporated after GSK-3 activation at residues 188, 184, 180, and 176.

Phosphorylation of N in the SR-rich region changes the consistency of phase-separated droplets with RNA from dense gel-like droplets to dynamic liquid-like droplets (19). To determine the molecular basis for this process, we sought to identify the effect of phosphorylation on the structure of linker alone and in context of the CTD. The SR-rich region preceding the self-associating LRH is a stretch of 40 residues containing 7 arginine and 14 serine residues that are targets for phosphorylation (45). We used genetic code expansion to incorporate a single phosphoserine at known priming position 188 (pSer188) (46). Spectra of ^15^N-HSQC of pSer188 N_175–245_ and WT N_175–245_ are essentially identical, except for the resonances corresponding to residues proximal to the phosphorylation site (Fig. 2). Comparison of ^15^N R_1_, ^15^N R_2_ and {^1^H}-^15^N NOE revealed similar dynamic properties, with a modest increase in {^1^H}-^15^N NOE near the phosphorylation site and a modest decrease in R_2_ in the alpha helical region. Since phosphorylation typically forms a hydrogen bond between the phosphate and the backbone amide causing the strong downfield shift in the amide proton resonance for pSer188 and is expected to dampen fast motion, it is not surprising to see a modest impact on the {^1^H}-^15^N NOE in the region proximal to the site of phosphorylation. The chemical shift changes for residues 186, 187, 189, and 190 suggest some minor structural changes localized to the vicinity of S188.

Even though only a modest change in chemical shifts is observed in pSer188 N_175–245_ spectra, a major increase is observed in the peak intensities of the self-associating LRH that is also reflected in the decrease in R_2_. NMR spectra collected at decreasing concentrations like those of the WT N_175–245_ showed higher intensities for the helical resonances implicated in self-association, compared to WT N_175–245_ at similar concentration (Fig. 3). SV-AUC analysis of pSer188-N_175–245_ GFP also indicated weakened self-association (Fig. 3). Concentrations of 200 μM and 100 μM of pSer188-N_175–245_ GFP showed a single peak in the c(s) plots, with sedimentation coefficients of ∼3.3 and ∼3.1S, respectively, indicating a weaker self-association when compared to WT N_175–245_ which showed two peaks. SE-AUC of pSer188 N_175–245_ GFP determined a Kd for dimer dissociation of 235 μM, significantly weaker than the 77 μM of WT N_175–245_ (Fig. 3).

pSer188 N_175–365_ displayed similar behavior to WT in ^15^N HSQC spectra at decreasing concentrations (Fig. S3). As with WT, resonances corresponding to residues 216 to 232 were not detectable at concentrations as low as 10 μM (Fig. S4), indicating that a single phosphoserine incorporation is not sufficient to dissociate the LRH in the context of the CTD. We then tested if hyperphosphorylation would dissociate the LRH. Addition of GSK-3 (+GSK N_175–365_) which phosphorylates serine or threonine residues N-terminal of an initial site of phosphorylation (42). While GSK-3 will canonically result in four phosphoserines, recent MS analysis of the SR-rich linker primed at S188 and subsequently reacted with GSK-3 showed partial phosphorylation up to six sites (47). These results are consistent with our observed NMR spectrum of GSK-3–treated N_175–365_, which showed three strong phosphoserine resonances and additional weaker resonances corresponding to lower abundance phosphorylation of additional serines. ^15^N HSQC experiments at decreasing concentrations revealed that resonances corresponding to residues 216 to 232 re-appeared at concentrations as high as 100 μM, indicating that hyperphosphorylation prevents LRH self-association in context of the CTD (Fig. 4). The dissociation of the tetramer is confirmed by SV-AUC experiments, in which there is a single broad peak centering around ∼2.7 S (which corresponds to dimeric +GSK N_175–365_) at 340 μM and 200 μM.

### Phosphorylation decreases RNA binding in the linker region

**Figure 5:**
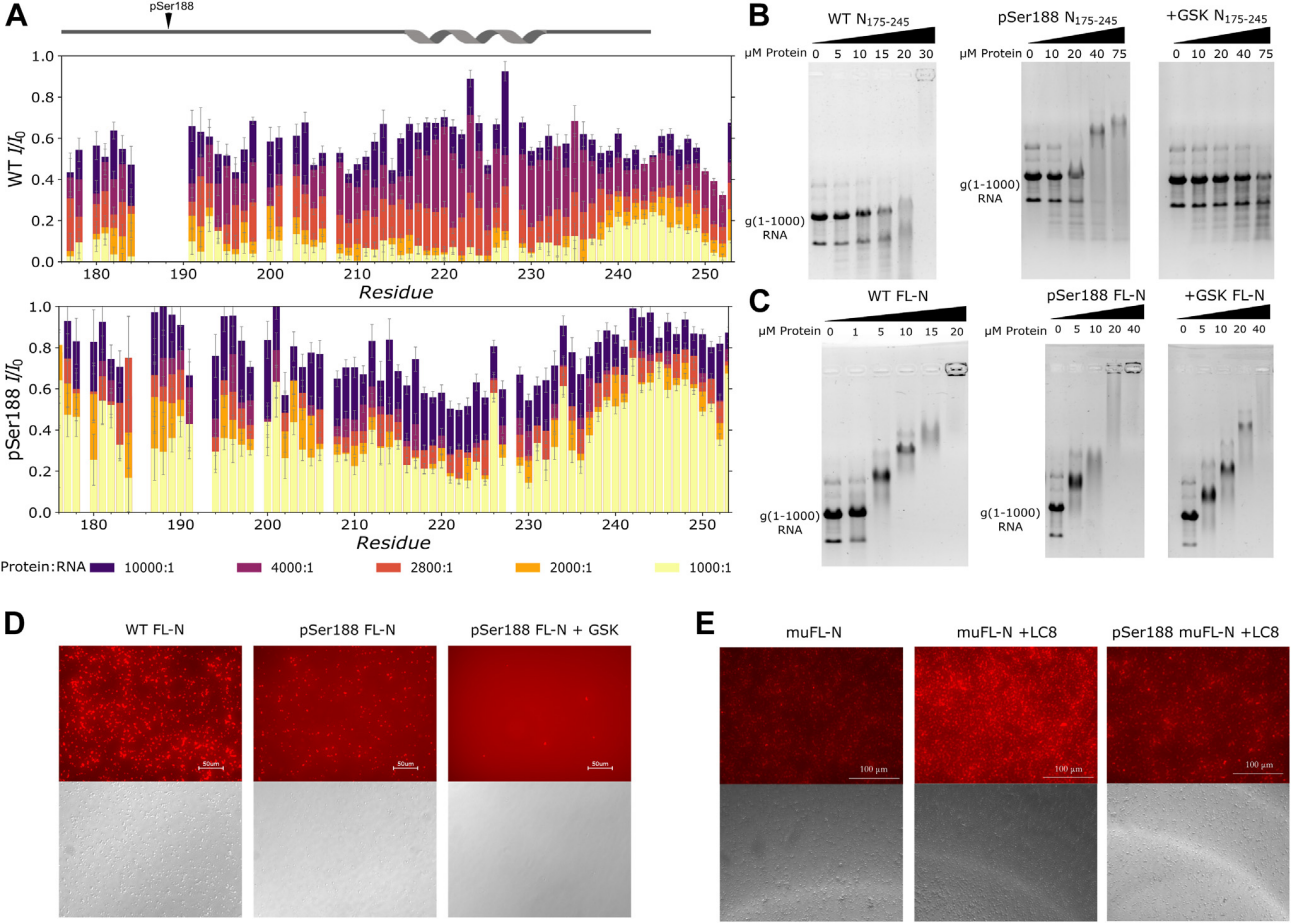
**Phosphorylation modulation of N-RNA interactions.***A*, peak intensity ratios based on NMR titrations of g(1–1000) RNA into ^15^N-labeled N_175–245_ (*top*) and pSer188 N_175–245_ (*bottom*). The protein:RNA ratio varied from 10000:1 to 1000:1. Data are plotted as intensity ratios for peaks with and without RNA. *B*, EMSA gels for WT N_175–245_ (0–30 μM) and pSer188 N_175–245_ (0–75 μM) with g(1–1000) RNA (0.5 μM). GSK-3 was used to hyperphosphorylate (+GSK) the pSer188 N_175–245_ (−GSK). *C*, EMSA gels for WT FL-N (0–20 μM) and pSer188 FL-N (0–40 μM) with g(1–1000) RNA (0.5 μM), along with GSK-3 hyperphosphorylation. For (B) and (C), the g(1–1000) RNA migrates as multiple bands because the RNA is not denatured and can adopt secondary structures. *D*, WT FL-N (*left*), pSer188 FL-N (*middle*), and hyperphosphorylated FL-N (+GSK FL-N) (*right*) liquid-liquid phase separation (LLPS) using fluorescence imaging (*top*) and bright field (*bottom*). The g(1–1000) RNA was labeled with cy3 for fluorescence imaging. Images were taken at 40× magnification, and the scale bar represents 50 μm. All LLPS experiments were collected at 37 °C after 2 h of incubation. *E*, LLPS utilizing the same conditions as (*D*) for muFL-N, muFL-N with LC8 added, and pSer188 muFL-N with LC8 added (from *left* to *right*, respectively). The scale bar represents 100 μm. FL-N, full length N protein; GSK, glycogen synthase kinase 3β; LC8, dynein light chain 8.

RNA binding to N is primarily driven by interactions with the NTD and, to a lesser extent, the CTD (24, 28). The central linker region shows direct interactions with RNA in the presence of the NTD (30). Here, we confirm using EMSA that N_175–245_ corresponding to the central linker alone can directly interact with the first 1000 nts of the 5’-end of SARS-Cov-2 viral RNA (g(1–1000)) (Fig. 5). Increasing concentrations of N_175–245_ caused an increasing shift of the g(1–1000) to a higher molecular weight, indicating that the protein is binding the RNA and reducing its mobility in the gel in a concentration-dependent manner.

We utilized NMR to probe the RNA interaction sites within N_175–245_. Addition of increasing concentrations of g(1–1000) RNA to ^15^N-labeled WT N_175–245_ caused most of the resonances to disappear, consistent with the formation of high molecular weight complexes. Resonances that are still observed at a 1000:1 protein:RNA molar ratio are localized to the C-terminal end of the WT N_175–245_ containing the residual tobacco etch virus (TEV) cleavage site, indicating these residues are not significantly interacting with g(1–1000) (Fig. 5). The significant line broadening of the remaining resonances suggests that the SR-rich region and the LRH are interacting with RNA, with only modest differences between the regions. This observation is consistent with a previous report that shows peak attenuation in this region when using a small piece of RNA (48).

We next determined if phosphorylation altered RNA binding using pSer188 N_175–245_. We found that the pSer188 N_175–245_ also bound RNA, based on EMSA analysis, but with weaker affinity (Fig. 5). Weaker binding was also demonstrated by NMR titrations of ^15^N-labeled pSer188 N_175–245_, with g(1–1000) RNA showing less peak attenuation at similar concentrations of RNA compared to the WT N_175–245_ (Fig. S5). Residues 175 to 200 also show less peak attenuation, suggesting that the double negative charge of pSer188 is sufficient to counter the favorable electrostatic interactions between the RNA and the arginine-rich linker. In contrast to the WT, the LRH resonances are more attenuated in the pSer188 N_175–245_ spectra with RNA, indicating that the RNA could still weakly interact with the helical region or that the RNA is altering the monomer-dimer exchange leading to additional broadening in the helical region.

Having observed a modest impact on RNA binding from a single phosphorylation, we next examined the impact of hyper-phosphorylation on RNA binding using GSK-3. Our EMSA results on GSK-3–hyperphosphorylated pSer188 N_175–245_ show full disruption of the interaction between N_175–245_ and g(1–1000) RNA even at 75 μM protein.

### Phosphorylation of FL-N attenuates LLPS but not RNA binding

Having found that phosphorylation attenuated binding of N_175–245_ to RNA, we performed similar experiments on the FL-N. In contrast to what we observed with N_175–245_, EMSA analysis showed that RNA-binding affinity to the FL-N was not affected by phosphorylation and hyperphosphorylation (Fig. 5) clearly indicating that linker phosphorylation at these four sites does not significantly alter g(1–1000) RNA binding by FL-N.

Phosphorylation has also been consistently shown to alter the phase-separation properties of FL-N (11, 19). We thus tested the effect of phosphorylation on LLPS behavior in the context of the full-length protein by comparing the ability of pSer188 and WT FL-N proteins to form droplets. WT FL-N phase separated at 4 μM protein with 50 nM g(1–1000) RNA at 37 °C (Fig. 5 and Table S1). The droplets formed with pSer188 FL-N protein were on average similar in size to WT FL-N but much fewer in number. The difference became even more pronounced in the GSK-hyperphosphorylated protein which showed almost no phase separation under these conditions (Fig. 5), with only a handful of droplets formed, clearly demonstrating that phosphorylation in the SR-rich region inhibits LLPS. The size of droplets formed in WT compared to pSer188 FL-N are similar, but the number of droplets formed by hyperphosphorylated FL-N was ∼70× less than the singly phosphorylated pSer188 FL-N and 180× less than the number of droplets observed for WT FL-N (Table S1).

### A switchable self-association domain to dissect effect of phosphorylation on LLPS

To test the contribution of the self-associating LRH to LLPS, we engineered a variant of FL-N with a switchable self-association capability by replacing the LRH with a disordered 10 amino acid segment containing a TQT recognition motif (KAIDAATQTE) specific for the dimeric hub protein LC8. LC8 is a dimerizing hub as it binds two disordered chains of partner proteins at the TQT motif (49). By replacing the LRH with a TQT motif of similar length, we have created a construct of FL-N (muFL-N) that will only self-associate in the linker in the presence of LC8 but is quite disordered without. We tested that LC8 indeed binds muFL-N (Fig. S6) and forms a tight complex. LLPS experiments on muFL-N showed less droplet formation with g(1–1000) RNA when compared to WT FL-N at similar conditions (Fig. 5), but upon addition of LC8, muFL-N showed extensive phase separation with g(1–1000) RNA (Fig. 5 and Table S2). Addition of LC8 to phosphorylated muFL-N (pSer188 muFL-N) increased LLPS but not to the same extent as in the absence of phosphorylation (Table S2 and Fig. 5), indicating that disrupting self-association in the linker is important but is not the only determinant of reduced LLPS. These data implicate the LRH as a primary modulator of LLPS as in both phosphorylated and unphosphorylated muFL-N LLPS experiments, LC8-induced dimerization promotes phase-separation behavior.

## Discussion

The structure of N is generally depicted with two folded domains, NTD and CTD, that are dimerized by the CTD and linked by a central disordered linker containing an α-helical region, LRH (Fig. 1). At the N terminus of the disordered linker is a positively charged SR-rich region that can undergo hyperphosphorylation. Given that the SR region is hyperphosphorylated in infected cells but unphosphorylated during viral assembly in infectious virions, it is significant to understand the molecular processes that drive this switch. Here, we show that the LRH-spanning residues 216 to 232 forms self-associated dimers and higher order aggregates in the unphosphorylated form, but its self-association is significantly weakened by phosphorylation and hyperphosphorylation at sites within the SR-rich region about 30 amino-acids distant from the LRH. Phosphorylation also attenuates RNA binding to the SR region, but while it does not significantly affect RNA binding to the full-length protein, it drastically alters its LLPS.

### Higher order association in FL-N is driven by the LRH-forming dimer of dimers

Since the crystal structure of the CTD was solved for a dimer (21, 23), it has been recognized that the CTD alone is the dimerization domain of N. Importantly, the CTD is a stable dimer and does not form higher order oligomers at NMR concentrations (22, 24). Work in the literature based on AUC and molecular dynamics reported a higher order trimeric coiled-coil mediated by intermolecular interactions in the LRH (34). Also reported is a mutation in N at the beginning of the LRH, G215C, exhibiting substantially stronger self-association and shifting self-association to a tetrameric oligomeric state (33). Using sedimentation equilibrium AUC on a construct containing the LRH and the CTD, we indeed identified a higher order species in addition to the dimer at concentration above 100 μM (Fig. 4). We assign this oligomerization state to a tetramer rather than a trimer for the following reasons: the LRH self-association is coupled to CTD dimerization and therefore the dimer is always the more stable species, and at higher concentration, it will populate a dimer of dimers or a tetramer 4-helix bundle that brings two dimers of N together. In this model, the LRH cannot dissociate in the presence of the CTD dimer but forms an intra dimer helix–helix interaction at low protein concentration, while the 4-helix bundle tetramer forms at high concentration. Multivalent binding of RNA to N will increase the effective local concentration shifting the equilibrium towards a tetramer or higher.

To deconvolute the contribution of the LRH from that of the CTD to formation of the higher order structure, we used NMR and AUC on constructs of the linker alone and the linker plus CTD and compared these results to the CTD alone. LRH in the linker construct has a weak self-association of 77 μM which is significantly enhanced to about 1 μM in context of the CTD and can also form weak tetramers. A construct corresponding to the CTD alone is a stable dimer at all concentrations. Therefore, these studies show clearly that the LRH is required to form higher order assemblies but forms only multiples of dimers.

### Phosphorylation decreases LLPS formation by inhibiting LRH self-association

MD simulations suggested that phosphorylation of the SR region increases inter- and intra-peptide interactions through phosphate-arginine salt bridges (50). Phosphorylation of an SR region’s synthetic peptide also exhibited reduced binding to polyU RNA as well as showed more liquid-like droplets (50). More recently, hyperphosphorylation was reported to promote direct binding of the phosphorylated SR region to the RNA-binding domain of the NTD and thus providing a mechanism for inhibiting RNA binding (51). The reported work, however, did not address the effect of phosphorylation on the LRH, as we do below.

Our NMR results show that phosphorylation at serine 188 did not dramatically alter the monomeric structure of the linker. Instead, phosphorylation resulted in a lower propensity of the linker to self-associate, with a Kd, three-fold weaker than WT. To place these results in context of the full-length protein, we determined the effect of phosphorylation in the presence of the dimeric CTD which significantly enhances dimerization of the LRH. A single phosphorylation event did not affect LRH self-association, but hyperphosphorylation with GSK-3, which places a pSer at 3 to 6 sites, caused dissociation of the tetrameric form suggesting that the level of phosphorylation can be tuned in response to the level of the protein concentration and self-association needed.

How phosphorylation in the SR region, about 30 amino-acid residues distant from the beginning of the LRH, promotes its dissociation is not entirely obvious. Comparison to other RNA-binding proteins with SR-rich regions gives some clues and suggests that phosphorylation could simply be increasing the solubility of the protein, preventing oligomerization (45). Phosphorylation significantly alters the net charge of a protein, adding a −2 charge with each phosphorylated residue at physiological pH. In TDP-43, for example, phosphorylation was proposed as a preventative cellular mechanism against aggregation (52). TDP-43 hyperphosphorylation was also shown to reduce phase separation and aggregation. One attractive explanation is that serine residues are more prone to interact with other protein residues than with solvent, while phosphoserine would interact more with water molecules than be involved in protein–protein interactions (45). Increased interactions with water would still not explain our observation of phosphorylation promoting dissociation 30 residues away, however. It is likely that solvation would change the structure of the SR region from a more extended to flexible with local structures; a change supported by chemical shift differences and a slight increase in the heteronuclear NOEs in the phosphorylated form. Local structures could interfere with stacking of the LRH in a helical bundle, thus opposing higher order formation.

### Phase separation and RNA binding

While SR-rich regions of proteins are often involved in RNA interactions (53, 54), in SARS-CoV-2, the NTD is the primary RNA-binding domain, but the CTD and the disordered domains are all capable of interacting with RNA with varying affinity and specificity. We show here direct interaction between the SR region and RNA, but its phosphorylation resulted in reduced RNA binding, while hyperphosphorylation by GSK-3, primed with pSer188, dramatically abolished it. Interestingly, in contrast to the isolated linker domain, WT, pSer188, and hyperphosphorylated FL-N all interact strongly with RNA as shown by EMSA (Fig. 5). These results demonstrate that while phosphorylation of the linker inhibits RNA binding at the SR-region, it does not inhibit overall RNA binding by FL-N presumably because phosphorylation would leave the high affinity NTD-binding site unaltered. A recent preprint reported a direct interaction of the phosphorylated region with the RNA site on the NTD, potentially blocking RNA binding and abolishing binding to FL-N (51). We see no effect on RNA binding with the GSK-phosphorylated FL-N. The discrepancies between these studies and ours could be explained as due to their use of a short RNA sequence while ours uses a 1000 nt viral RNA. The 1000 nt RNA may have some allosteric effects preventing the blocking of the NTD. Further, a larger RNA with multivalent sites will compete with any weak interactions between the SR-rich region and the RNA-binding region of the NTD.

Exciting about our findings is the effect of phosphorylation on phase separation of FL-N. Phosphorylation by a combination of CDK-1 and GSK-3 was reported to alter phase-separation behavior, inducing a transition from gel-like to liquid-like droplets (19). Our GSK-hyperphosphorylated FL-N shows significant reduction in both the size and number of droplets formed. Interesting to note is that while RNA interaction is certainly required for phase separation, phase-separation behavior can be inhibited by phosphorylation in the disordered linker region even while overall RNA binding remains intact.

### A model for how hyperphosphorylation in the SR region attenuates phase separation

**Figure 6:**
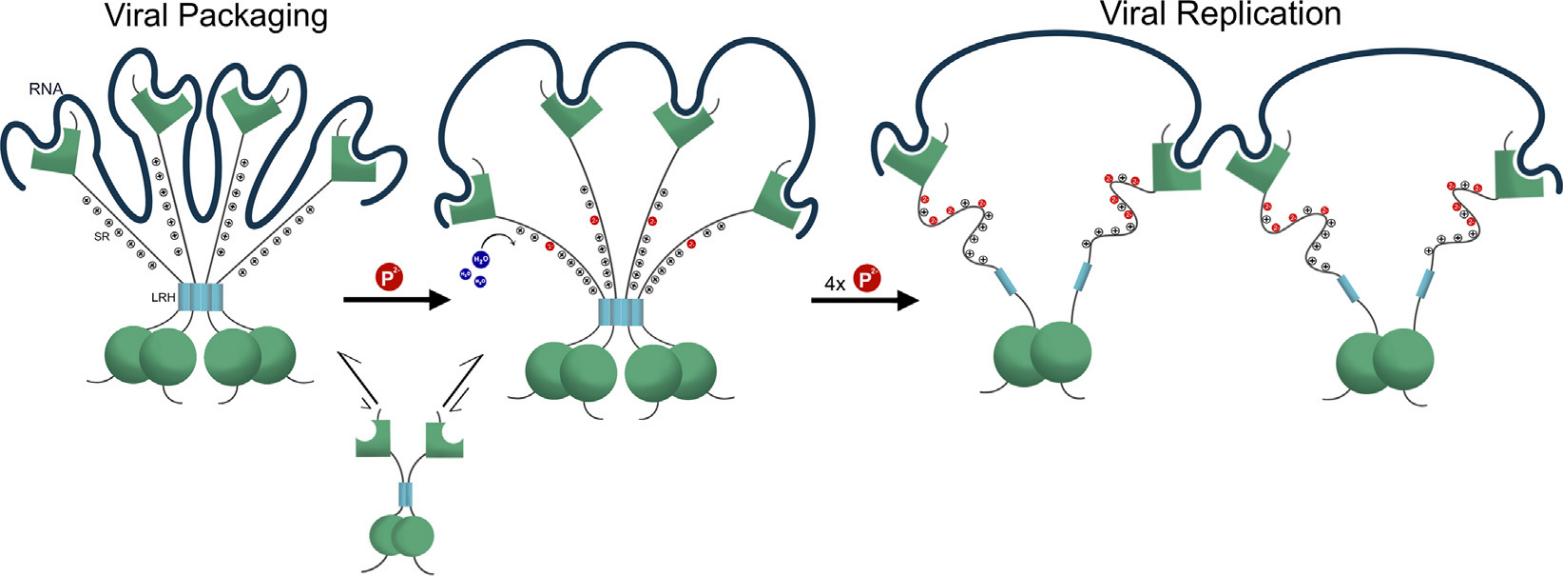
**Proposed model of how phosphorylation acts as a molecular switch for the role of N from viral packaging to viral replication.** FL-N is depicted here using similar illustration as in Figure 1C. (*Left*) Unphosphorylated FL-N is in a dimer-tetramer equilibrium which shifts to tetramer when bound to RNA. The positive charges in the SR-rich region cause elongation due to charge–charge repulsion. RNA intercalates by binding to the SR region of the linker, resulting in a compacted RNA expected to be most populated in LLPS and in viral packaging. (*Middle*) A single phosphorylation event (*red*) introduces negative charges in the SR causing some structural change. RNA remains bound to the NTD but does not bind the linker resulting in reduced compaction. (*Right*) Four phosphorylation events with GSK-3 causing significant structural changes in the SR-region of GSK hyperphosphorylated FL-N and dissociation of the 4-helix bundle tetramers. The resulting structure of FL-N is a dimer with significant flexibility in the linker after dissociation of the LRH causing significant dispersing of the RNA, consistent with the model expected in viral replication. Two N proteins are shown to illustrate multivalent binding. Phosphates are indicated by *red circles*, and arginines by circled pluses. RNA is depicted by the *dark blue line*. FL-N, full length N protein; GSK-3, glycogen synthase kinase 3β; NTD, N-terminal domain; SR-rich, serine/arginine-rich.

The model of Figure 6 summarizes our data and proposes a process of how phosphorylation switches the role of N from viral packaging to viral replication. The N protein is in a dimer-tetramer equilibrium that shifts towards tetramer at high protein concentration as shown here or forms higher order oligomers when bound multivalently to RNA (55). It is well established that viral packaging would involve genome compaction through multivalent protein:RNA and protein:protein interactions (56). In the absence of phosphorylation, the LRH self-association organizes the SR regions in an elongated manner primed for higher order aggregation when bound to RNA. A single phosphorylation introduces repulsive charges that disrupt charge patterning which is normally associated with LLPS (57) preventing stacking of the SR regions and improving solubility thus reducing LLPS while keeping the self-association of LRH. The local effect of phosphorylation is confirmed by using the switchable dimer which shows that self-association is not enough to restore full LLPS. Hyperphosphorylation is required to amplify the disruption in the SR regions and significantly introduces bound water molecules to cause dissociation of the LRH about 30 amino acids downstream, disrupting LLPS.

The model of Figure 6 also explains how abolishing binding of the SR region to RNA reduces compaction while keeping the RNA bound in the full-length protein. Binding to the linker intercalates the RNA tightly in the nucleocapsid during packaging and this is reversed upon phosphorylation. Dissociation of the LRH will increase its accessibility to other binding partners during viral maturation such as the viral protein nsp3a (58) and the host protein 14-3-3 (59).

## Concluding remarks

Phosphorylation in SR-rich proteins has been observed to increase solubility or disrupt alpha helical regions inhibiting their ability to self-assemble in fibrils (60). In TDP-43, phosphorylation of few key residues disrupts the helical structure and regulates condensate formation (61). Interesting to point out here, however, that in these examples, the phosphorylation effect is spread locally. The effect of phosphorylation of the N protein and its binding to RNA at multivalent sites is significantly more complicated. Our model presents an explanation of how phosphorylation in a disordered linker in addition to causing changes in the local environment can also act on the self-association of a distant helix that is easily responsive to long range site-specific changes, contributing to switching from genome replication to virus maturation.

## Experimental procedures

### Plasmid construction

The N_175–245_ plasmid construct was prepared by inserting DNA encoding amino acid residues 175 to 245 into a pRBC SUMO vector with a N-terminal bdSUMO tag, a C-terminal TEV protease cleavage site linked to GFP, and a hexahistidine tag (62). N_175–365_ plasmid construct was prepared by inserting DNA encoding amino acid residues 175 to 365 into a pRBC vector with a C-terminal TEV protease cleavage site linked to a hexahistidine tag. pSer188 N_175–245_ plasmid was generated as previously described (46). pSer188 N_175–365_ was prepared from the N_175–365_ plasmid by mutating S188 to an amber stop codon (TAG). pSer188- FL-N plasmid was generated by cloning the FL-N sequence into the pRBC vector, then mutating S188 to a TAG for expression with genetic code expansion. muFL-N and pSer188 muFL-N plasmids were prepared from the FL-N and pSer188-FL-N plasmids respectively, using site-directed mutagenesis to remove the DNA-encoding residues 216 to 232 and replace them with DNA encoding the sequence KAIDAATQTE, a sequence known to bind LC8 strongly (63).

### Protein expression and purification

To prepare WT N_175–245_ or WT N_175–365_ proteins, BL21(DE3) *E. coli* transformed with the appropriate plasmid were grown in LB-rich media to an OD of 0.6 to 0.8, then induced with 1 mM IPTG at 37 °C for 6 h. Stable isotope-labeled samples of WT N_175–245_ or WT N_175–365_ were expressed and induced using the same procedure as natural abundance, expect that the cells were grown in MJ9 minimal media supplemented with ^15^N ammonium chloride and ^13^C glucose as the sole nitrogen or carbon source as appropriate. Following induction, cells were harvested by centrifugation and either used immediately or stored at −80 °C.

WT N_175–245_ and WT N_175–365_ were purified using the TALON His-tag purification protocol (Clontech Laboratories). Cell pellets were lysed in 50 mM tris, 500 mM NaCl, 5 mM imidazole, 1 mM NaN_3_, pH 7.5 (high salt buffer) by sonication and centrifuged at 27,200 relative centrifugal force to remove cell debris. The supernatant was mixed with resin for 1 h and washed with 20 column volumes of high salt buffer followed by four column volumes 50 mM tris, 150 mM NaCl, 5 mM imidazole, 1 mM NaN_3_, pH 7.5 (low salt buffer). For WT N_175–245_, the SUMO solubility tag was cleaved with 200 nM SENP1 protease (46, 64, 65) (∼1:250 protease:protein) for 1 h at 4 °C on the resin. Proteins were eluted off the resin with high salt buffer supplemented with 300 mM imidazole. Proteins were exchanged into buffer containing 50 mM Tris, 300 mM NaCl, 5 mM imidazole, pH 7.5 using a PD-10 desalting column (Cytiva). The tag was cleaved by incubating overnight with TEV protease (66) (∼1:20 protease:protein) at 4 °C and then removed by reverse affinity purification with Talon His-Tag resin. Proteins were concentrated and exchanged into 50 mM sodium phosphate, 150 mM NaCl, pH 6.5 (NMR buffer).

FL-N and muFL-N were prepared using a modified procedure based on one previously described for FL-N (22). The plasmid was transformed into *E. coli* Rosetta (DE3) cells, cultured in 2xYT media to an OD of 0.6, and induced with 1 mM IPTG at 18 °C overnight. Cells were harvested by centrifugation then resuspended in 50 mM sodium phosphate, 1M NaCl, 5 mM imidazole, 1 mM NaN_3_, 1 mg/ml lysozyme, pH 8.0 and incubated for 1 h at 4 °C. Cells were sonicated 3× 2 min and centrifuged at 27,200 relative centrifugal force for 45 min. The clarified lysate was mixed with Talon His-Tag resin, incubated for 1 h at 4 °C, and then washed with 20 column volumes of 50 mM sodium phosphate, 3M NaCl, 10 mM imidazole, 1 mM NaN_3_, pH 8.0 to remove nonspecifically bound RNA. The protein was eluted with 50 mM sodium phosphate, 300 mM NaCl, 350 mM imidazole, 1 mM NaN_3_, pH 8.0 FL-N, then concentrated, and further purified on a Superdex 200 column in 50 mM sodium phosphate, 150 mM NaCl, pH 7.5 buffer. The protein was concentrated and either used immediately or stored flash frozen at −80 °C.

To prepare pSer188 N_175–245_ and pSer N_175–365_, BL21(DE3) ΔSerB *E. coli* cells were transformed simultaneously with the appropriate expression plasmid and pKW2-EFsep. For natural abundance samples, cells were grown in 2xYT to an OD of 0.6 to 0.8 and induced with 1 mM IPTG for 48 h at 18 °C. Stable isotope-labeled pSer188 N_175–245_ and pSer N_175–365_ were expressed and purified as previously described (46). Briefly, cells were grown to an OD 0.6 to 0.8 in MJ9 minimal media supplemented with ^15^N Celtone (0.2%) and then induced with 1 mM IPTG for 48 h at 18 °C. Cells were harvested by centrifugation and stored at −80 °C. Protein was purified using the same method as used for the WT samples above, except that buffers were supplemented with phosphatase inhibitors (10 mM NaF, 2.5 mM sodium pyrophosphate, and 1 mM orthovanadate).

To prepare pSer188 FL-N and pSer188 muFL-N proteins, the appropriate expression plasmid was transformed simultaneously with pKW2-EFsep into BL21(DE3) ΔSerB *E. coli* cells. Cells were grown in rich-Auto-inducing Media (37) at 37 °C until OD 600 reached 1.3 and then at 18 °C for 48 h. Cells were harvested and the protein was purified using the same methods as FL-N, except that the buffers were supplemented with phosphatase inhibitors (10 mM NaF, 2.5 mM sodium pyrophosphate, and 1 mM orthovanadate).

For hyperphosphorylated protein, 80 μM of target protein (pSer188 linker, pSer188 N_175–365_, FL-N pSer188, or muFL-N pSer188) was incubated with 80 nM of GSK-3 in a buffer containing 20 mM Tris pH 7.4, 150 mM NaCl, 10 mM MgCl_2_, and 1 mM ATP, at 37 °C for 20 h. Hyperphosphorylated proteins were further purified by size-exclusion chromatography using a Superdex S75 column. Phos-Tag SDS-PAGE gels were used to confirm the phosphorylation status of all phosphorylated samples.

LC8 expression and purification was performed as previously described (67). The GSK-3 protein was expressed and purified as previously described (46). Table S3 lists the final buffer conditions for each protein and experiment.

### NMR spectroscopy

NMR experiments were performed using an 800 MHz Bruker Avance III HD NMR spectrometer equipped with a triple resonance (TCI) cryogenic probe. Backbone resonance assignments were made using a suite of BEST triple resonance experiments, including HNCO, HNCACB, HNCACO, and HNCOCACB (68). All NMR data were processed (apodized, zero filled, Fourier Transformed, and phased) using nmrPipe (69) and analyzed in nmrviewJ (70). Three dimensional experiments were collected using nonuniform sampling, and nonuniform sampling artifacts were suppressed using SMILE (71). Resonance assignments were deposited in the BMRB under ascension number 51904. ^15^N nuclear spin relaxation parameters were measured using Bruker temperature compensated pulse sequences, with eight unique delay times. The 60 ms delay was collected in triplicate to aid with error estimation. Peak intensities were fit in nmrviewJ to an exponential decay model, with Monte Carlo–based error estimation. For the {^1^H}-^15^N NOE, the D1 delay was increased to 8 s to ensure complete relaxation between scans. NMR titrations of N_175–245_ with RNA were performed using 2D ^15^N-BEST-TROSY experiments. Peak intensities were measured in nmrviewJ and normalized to the corresponding peak in the spectrum without RNA. Secondary chemical shifts were calculated using sequence, temperature, and pH-corrected chemical shifts (72). NMR concentration titrations on N_175–365_ were performed using 2D ^15^N TROSY HSQC experiments. All experiments were conducted at 10 °C.

### Electrophoretic mobility shift assay

The first 1000 nucleotides from the viral genome (g(1–1000) RNA) at a final concentration of 0.5 μM was incubated with increasing concentrations of protein (range of 0–75 μM, in 20 mM Tris, 150 mM NaCl, 1 mM DTT, pH 7.5) at room temperature for 20 min. The total reaction volume was 10 μl. After incubation, 2 μl of 6× loading dye was added to the reaction before loading on a 1% agarose gel. The gel was run at 150 V for 1 h. RNA bands were stained with Midori Green Nucleic Acid staining solution (Bulldog Bio. Inc) and visualized using a Bio-Rad Gel Doc Image system.

### Analytical ultracentrifugation

Analytical ultracentrifugation was performed using a Beckman Coulter Optima XL-A analytical ultracentrifuge equipped with absorbance optics. SV-AUC experiments used either N_175–245_ protein tagged with GFP because N_175–245_ by itself does not have a strong UV absorbance or a construct of N_175–365_. All SV-AUC samples were run in standard 2-channel sectored cells using an An60-Ti rotor at 20 °C. The concentration of each protein was varied from 50 to 200 μM. Samples were spun at 42,000 rpm and with 300 scans per sample. Data were fit to the continuous c(s) model using SEDFIT (73). Buffer density and viscosity were calculated using SEDNTERP (74). For sedimentation equilibrium analysis, three concentrations of each sample were loaded into 6-well cells. Samples were centrifuged at 10,000, 13,000, and 18,000 rpm in an An60-Ti rotor for 30 to 36 h at each rotor speed. For GFP N_175–245_, data were fit to either a single ideal species or monomer-dimer equilibrium using the software Heteroanalysis (https://colelab.uconn.edu/) (75). For N_175–365_, data were fit to a dimer-tetramer model.

### LLPS and microscopy

Fluorescence microscopy images were taken on a Keyence BZ-X700/BZ-X710 microscope with a 40× objective lens and a 384-well plate (Cellvis P384-1.5H-N); images were processed using BZ-x viewer and BZ-x analyzer software (https://www.keyence.com/landing/microscope/lp_fluorescence.jsp). For this experiment, cy3-labeled RNA was diluted into nuclease-free water to reach a final concentration of 50 nM when added to the protein sample. Stocks of unlabeled protein were prepared by diluting into 20 mM Tris, 150 mM NaCl, 1 mM DTT, pH 7.5 droplet buffer. For FL-N constructs, samples were prepared at a total concentration of 4 μM. For linker constructs, samples were prepared at a total concentration of 20 μM. Protein samples were prepared by combining 27 μl of protein stock of the appropriate concentration with 3 μl of cy3-labeled g(1–1000) RNA for a total sample volume of 30 μl. For muFL-N samples with LC8 added, 3.6 μM LC8 was combined with muFL-N and cy3-labeled g(1–1000). The samples were incubated at 37 °C for 2 h and subsequent imaging was taken. Foci were counted using ImageJ software (https://imagej.net/ij/). Average foci diameter was calculated in ImageJ from a random sampling of 10 foci from each image.

## Data availability

All data needed to evaluate the conclusions in the paper are present in the paper and/or the supporting information.

## Supporting information

This article contains supporting information.

## Conflicts of interests

The authors declare that they have no conflicts of interest with the contents of this article.
