# Supplementary material for: Phosphorylation in the Ser/Arg-rich region of the nucleocapsid of SARS-CoV-2 regulates phase separation by inhibiting self-association of a distant helix
Source: J Biol Chem. 2024 May 7;300(6):107354. doi: 10.1016/j.jbc.2024.107354 (PMC11180338; doi:10.1016/j.jbc.2024.107354)

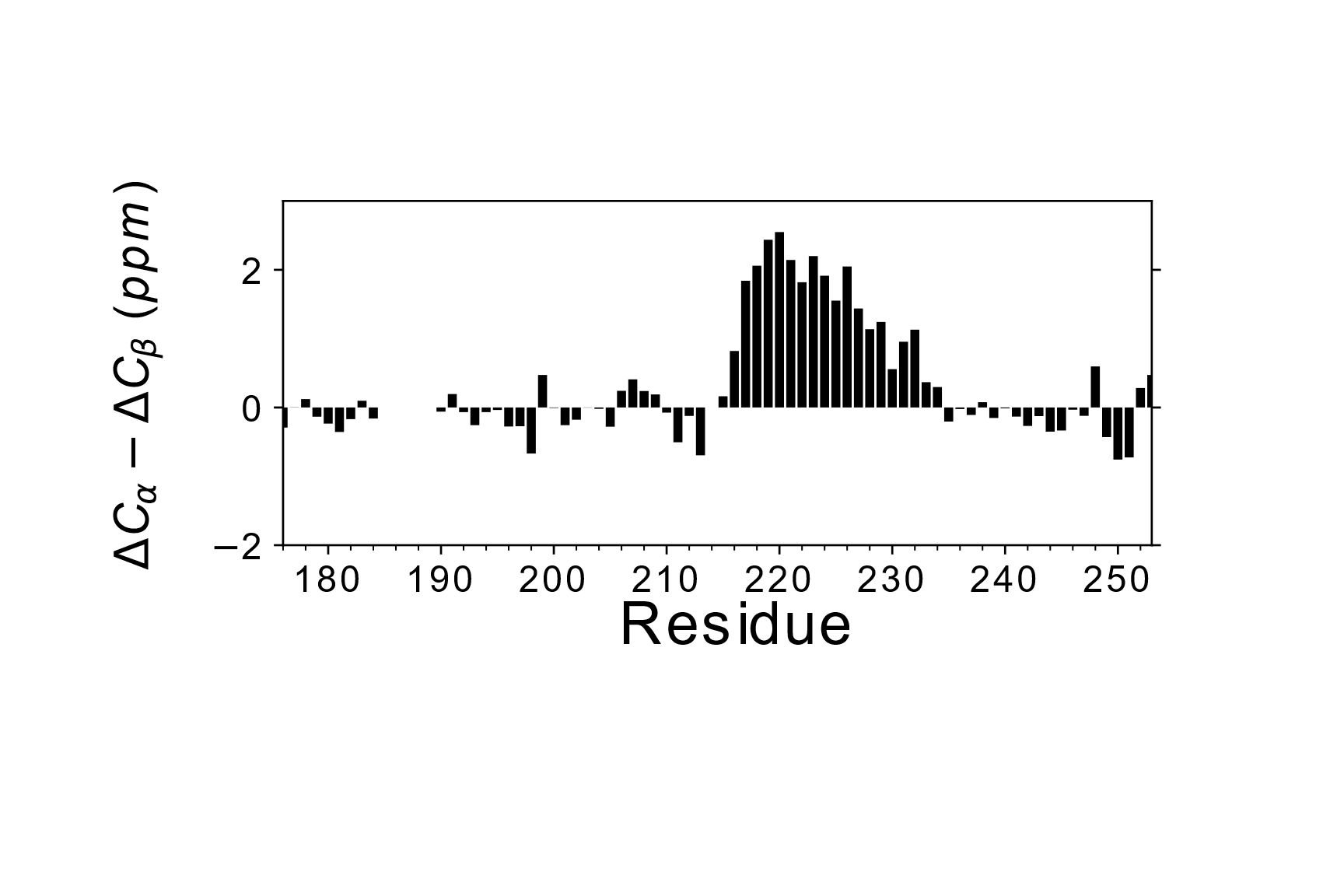


Fig. S1. Chemical shift indexing of WT N_175-245_. Significantly positive chemical shift values are between residues 216-232, indicating alpha helical structure.


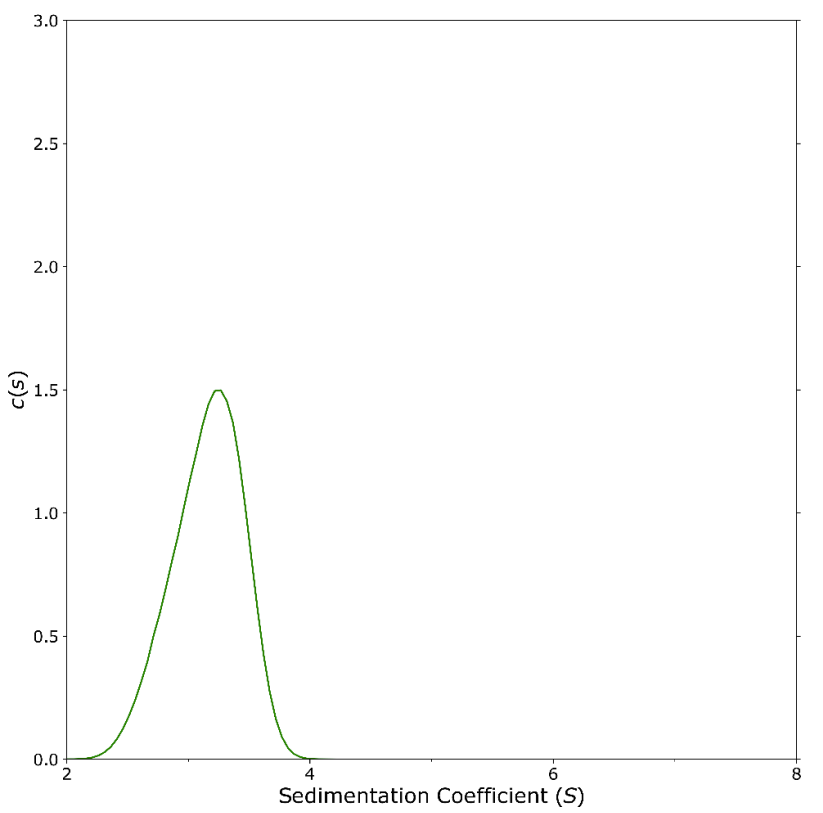


Fig. S2. SV-AUC of GFP. SV-AUC was performed on a sample of 200 μM GFP without N_175-245_. It showed a single peak with sedimentation coefficient of ~3.2 S, indicating no significant dimerization contribution from GFP under these conditions.


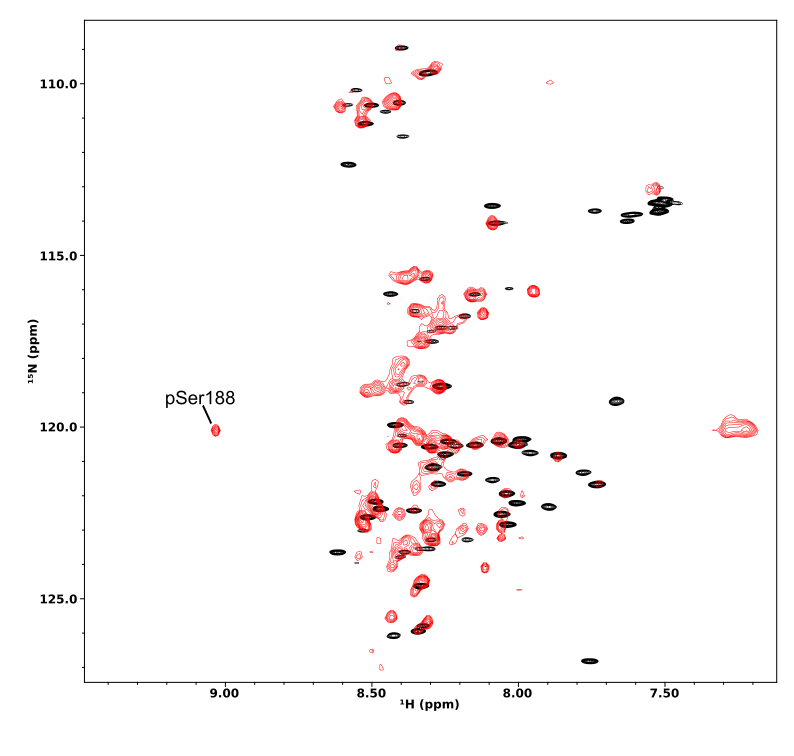


Fig S3. ^15^N-TROSY-HSQC spectra of 50μM pSer188 N_175-365_ (red) overlayed with WT N_175-245_ (black). Down-field resonance corresponding to phosphorylated serine is labeled. The absence of peaks in pSer188 WT N_175-245_ in the helical region confirms that a single phosphorylation event is not sufficient to inhibit self-association in the LRH.


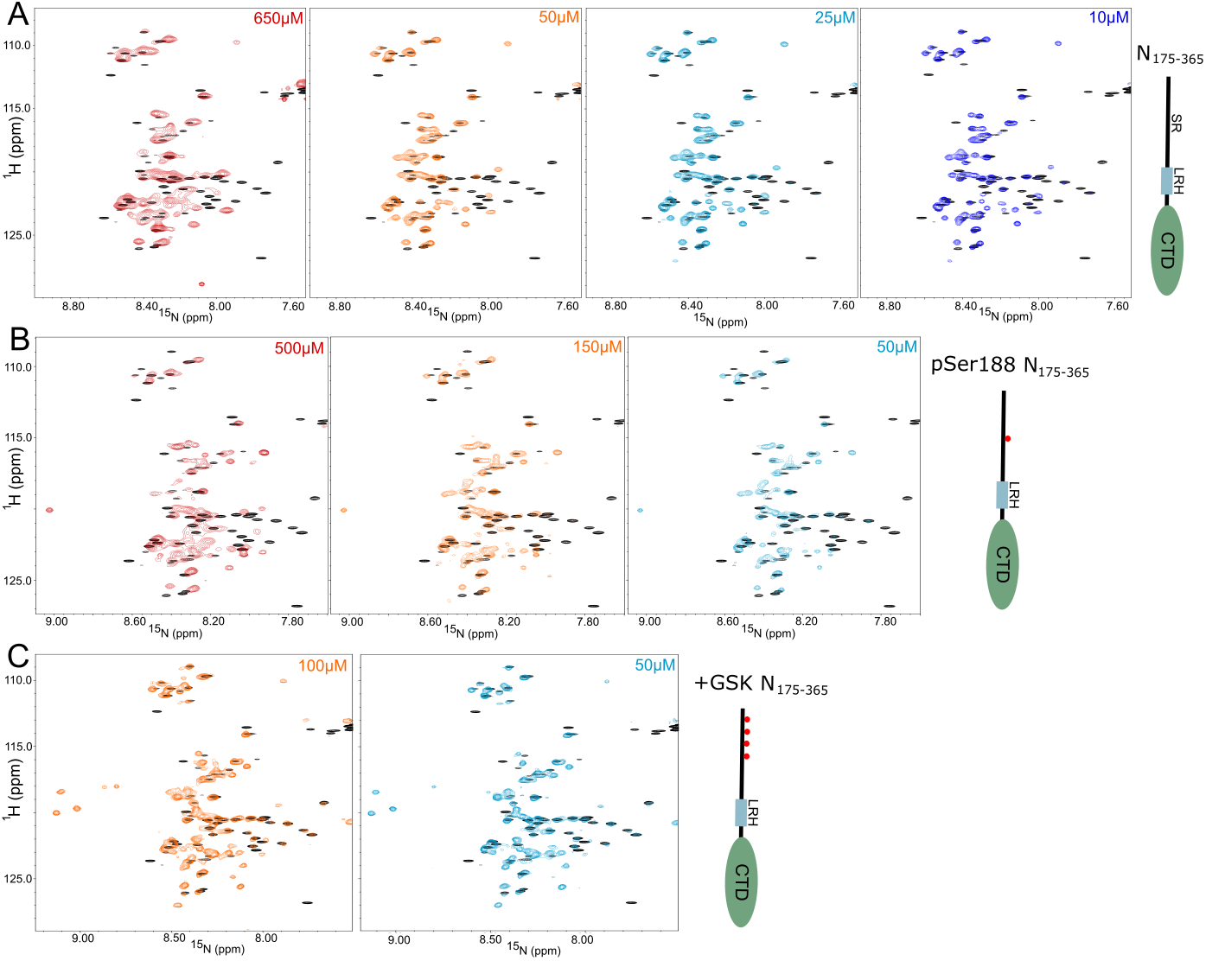


Fig. S4. ^15^N-TROSY-HSQC spectra as a function of concentration of N_175-365_ at varying phosphorylation levels. A. ^15^N-TROSY-HSQC spectra at decreasing concentrations of WT N_175-365_ from 650µM (left) to 10µM (right). B. 15N-TROSY-HSQC spectra at decreasing concentrations of pSer188 N_175-365_ from 500µM (left) to 50µM (right). C. 15N-TROSY-HSQC spectra at decreasing concentrations of +GSK N_175-365_ from 100µM (left) to 50µM (right). Resonances corresponding to helical residues 216-232 are not present in WT at the lowest concentration of 10µM and these residues are present in both concentrations of +GSK N_175-365._


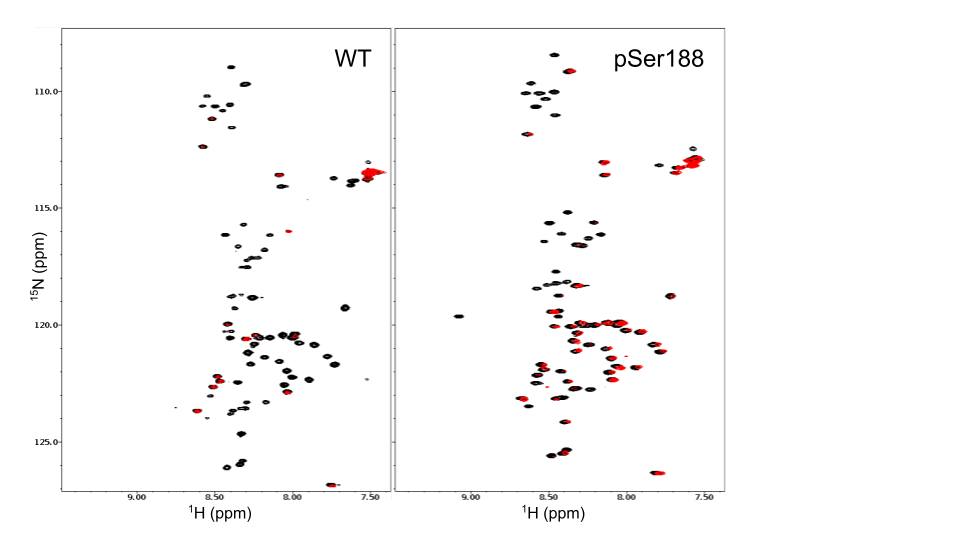
Fig. S5. Comparison of WT N_175-245_(left) and pSer188 N_175-245_ (right) ^15^N HSQC spectra of free (black) and bound (red) to the highest ratio of g(1-1000) to protein tested (1:1000). Many more peaks remain in the bound pSer188 indicating weaker binding.


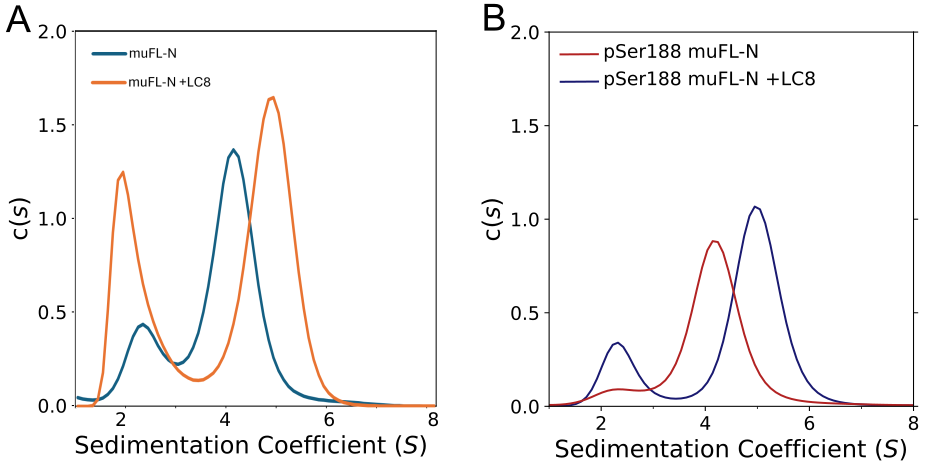


Fig. S6. Comparison of SV-AUC of muFL-N (A) and pSer188 muFL-N (B) with and without LC8 (1:1) showing a shift to higher S value in the presence of LC8 indicating binding.

Table S1. Droplet count and average diameter for each fluorescent image of LLPS experiments on WT, pSer188, and +GSK FL-N with g(1-1000). Droplet count was determined for the entire area of the image (~350µmx300µm) with ImageJ foci counting tool.

| **Construct** | **Count** | **Size (µm)** |
| --- | --- | --- |
| WT FL-N | 1080 | 3.3 |
| pSer188 FL-N | 402 | 3.2 |
| GSK FL-N | 6 | 3.5 |

Table S2. Droplet count for LLPS experiments on muFL-N, mu-FLN with LC8, pSer188 muFL-N, and pSer188 muFL-N with LC8, all with g(1-1000). Droplet count was determined for the entire area of the image (~350µmx300µm) with ImageJ foci counting tool.

| **Construct** | **Count** |
| --- | --- |
| muFL-N | 612 |
| muFL-N +LC8 | 912 |
| pSer188 muFL-N | 31 |
| pSer188 muFL-N +LC8 | 483 |

Table S3. Buffer conditions according to experiment.


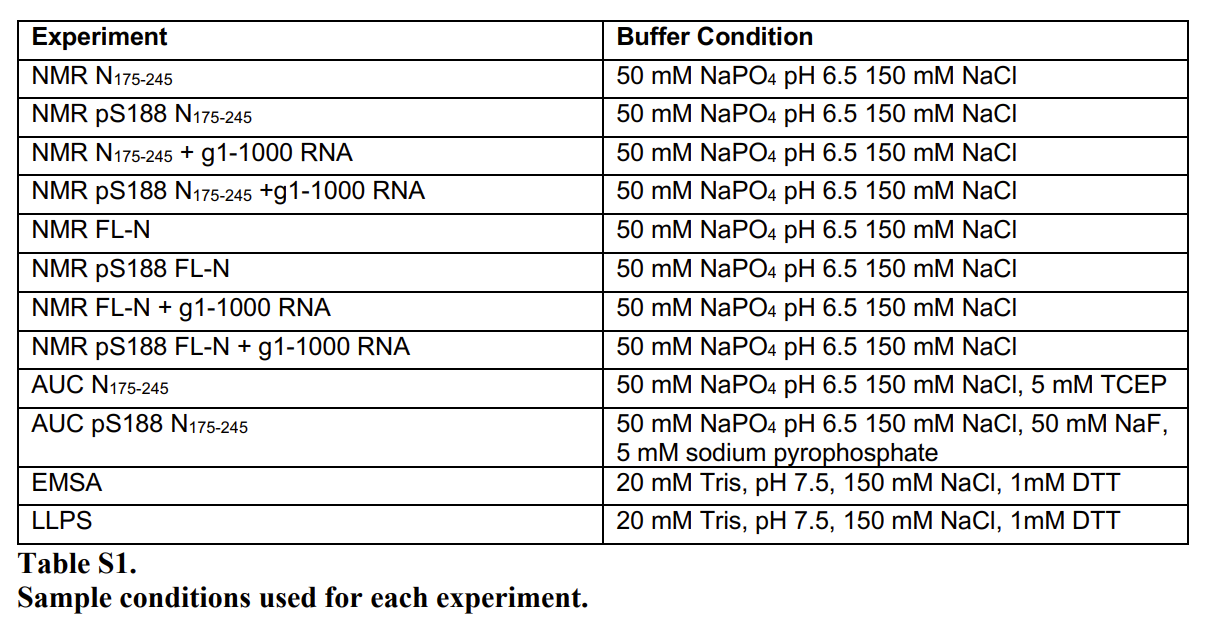

Supplement: Supplemental Figures S1–S6 and Tables S1–S3 [file mmc1.docx]
